# Long-Term Prospective Study of the Influence of Estrone Levels on Events in Postmenopausal Women with or at High Risk for Coronary Artery Disease

**DOI:** 10.1100/2012/363595

**Published:** 2012-06-04

**Authors:** Antonio de Padua Mansur, Tereza Cristina B. F. Silva, Julio Yoshio Takada, Solange Desirée Avakian, Célia Maria C. Strunz, Luiz Antonio Machado César, José Mendes Aldrighi, José Antonio F. Ramires

**Affiliations:** ^1^Heart Institute (InCor), University of São Paulo Medical School, 05403-000 São Paulo, SP, Brazil; ^2^School of Public Health, University of São Paulo, 01246-904 São Paulo, SP, Brazil

## Abstract

*Background*. The link between endogenous estrogen, coronary artery disease (CAD), and death in postmenopausal women is uncertain. We analyzed the association between death and blood levels of estrone in postmenopausal women with known coronary artery disease (CAD) or with a high-risk factor score for CAD. *Methods*. 251 postmenopausal women age 50–90 years not on estrogen therapy. Fasting blood for estrone and heart disease risk factors were collected at baseline. Women were grouped according to their estrone levels (<15 and ≥15 pg/mL). Fatal events were recorded after 5.8 ± 1.4 years of followup. *Results*. The Kaplan-Meier survival curve showed a significant trend (*P* = 0.039) of greater all-cause mortality in women with low estrone levels (<15 pg/mL). Cox multivariate regression analysis model adjusted for body mass index, diabetes, dyslipidemia, family history, and estrone showed estrone (OR = 0.45; *P* = 0.038) as the only independent variable for all-cause mortality. Multivariate regression model adjusted for age, body mass index, hypertension, diabetes, dyslipidemia, family history, and estrone showed that only age (OR = 1.06; *P* = 0.017) was an independent predictor of all-cause mortality. *Conclusions*. Postmenopausal women with known CAD or with a high-risk factor score for CAD and low estrone levels (<15 pg/mL) had increased all-cause mortality.

## 1. Background

Endogenous estrogens are associated with greater vascular protection in premenopausal women [1]. Estradiol is the main and most active representative of endogenous estrogens in premenopausal women. This protection occurs through genomic and nongenomic actions [2]. The antiatherogenic and antithrombogenic effects of estradiol slow the process of atherosclerosis, and therefore cardiovascular events occur later in women compared to men [3]. In postmenopausal women, a significant reduction in estradiol production occurs due to ovarian failure. The natural protection ceases to exist, and at this stage, a gradual and progressive increase begins in cardiovascular events in women. However, hormone replacement therapy with estrogen and progesterone was not effective in primary or secondary prevention of cardiovascular events in postmenopausal women [4, 5]. Several hypotheses have been considered to justify these results, among them, the characteristics of exogenous estrogens, the high dose used, the association of progestins, and time since menopause [6]. It is known, however, that in postmenopausal women production of endogenous estrogen is very low. In this period, estrone is the main representative of the endogenous estrogens and is produced by several tissues, especially adipose tissue. Estrone is the result of the process of aromatization of androstenedione that occurs in fat cells [7]. Obese women have higher plasma concentrations of estrone [8]. However, the influence of estrone on cardiovascular protection in postmenopausal women is unknown. A previous study showed higher plasma levels of estrone in obese patients with coronary artery disease (CAD) or at high risk for coronary heart disease [9]. The same study also showed that increased plasma levels of estrone were associated with a nonsignificant trend toward a lower incidence of cardiovascular events. However, the follow-up period was only 2 years. The current study analyzed, in the same population, the effects of serum estrone in death from all causes in a long-term followup.

## 2. Methods

### 2.1. Study Populations

Participants were 251 postmenopausal women aged 50 to 90 years who had known CAD or were at high risk for CAD based on a risk factor score of 4 or more points, using the score developed for use in raloxifene trials [10]. Patients using postmenopausal hormone therapy and with advanced stage or clinically limiting disease were not included in the study. Women were patients in the ambulatory care clinic of a tertiary cardiology hospital, who were followed from March 2004 to December 2010. Primary end-point was death of any cause. Follow-up data were obtained through telephone calls and validated if given by a family member. Five patients were missed.

### 2.2. Clinical Characteristics

Baseline clinical characteristics included body mass index (BMI), blood pressure, medication use, and history of cardiac procedures and cardiac events. Laboratory tests for biologic CAD risk factors included fasting venous blood glucose, triglycerides, total cholesterol, HDL-cholesterol, LDL-cholesterol, C-reactive protein, and estrone. Risk factors for CAD were diabetes mellitus (fasting blood glucose ≥126 mg/dL [11]); hypertension (diastolic blood pressure ≥90 mm Hg [12]); dyslipidemia (triglycerides ≥200 mg/dL and/or LDL cholesterol ≥130 mg/dL [13]); family history (CAD occurring in parents before age 55 for men, 65 for women and siblings); at least 6 months of daily smoking of any kind or quantity of tobacco [14]; obesity status based on BMI (kg/m^2^) defined by the National Institutes of Health and the World Health Organization as normal (≥18.5 to <25), overweight (≥25 to <30), and obese (≥30) [15].

### 2.3. Laboratory Measurements

Morning blood was collected after a requested 12-hour fast. Estrone samples were measured on frozen plasma by a manual processing radioimmunoassay method using DSL 8700 (Diagnostic Systems Laboratories, Inc., Webster, TX, USA) with reference values 14.1–102.6 pg/mL for postmenopausal women. Based on the lower limit of the reference value in this laboratory, low estrone levels were defined as <15 pg/mL (43 patients) versus ≥15 pg/mL (208 patients). High sensitivity C-reactive protein was measured using an immunoturbidimetric ultrasensitive detection method, (Roche Diagnostics GmbH, D-68298 Mannheim, Germany), with a normal reference value <0.5 mg/dL.

The ethics committee of the Heart Institute (InCor) approved the study design, and all participants provided written informed consent.

### 2.4. Statistical Analysis

A sample size of at least 200 patients, with 100 subjects per treatment group, was determined to give 80% power to detect a 20% difference in the incidence of cardiovascular events with a 5% significance level.

The chi-square test was used for analysis of categorical variables, and Student's *t* test or 2-way ANOVA analysis was used for continuous variables. Mortality analyses used Kaplan-Meier estimation curves stratified by low or normal estrone levels (<15 and ≥15 pg/mL, resp.). Three models of Cox multivariate regression analysis were done. Model 1 was adjusted for age, hypertension, body mass index, diabetes, dyslipidemia, and estrone; model 2 was adjusted for hypertension, body mass index, diabetes, dyslipidemia, and estrone; model 3 was adjusted for body mass index, diabetes, dyslipidemia, and estrone. The statistical software used was SAS 9.2 (SAS Institute Inc, Cary, NC, USA).

## 3. Results

**Figure 1:**
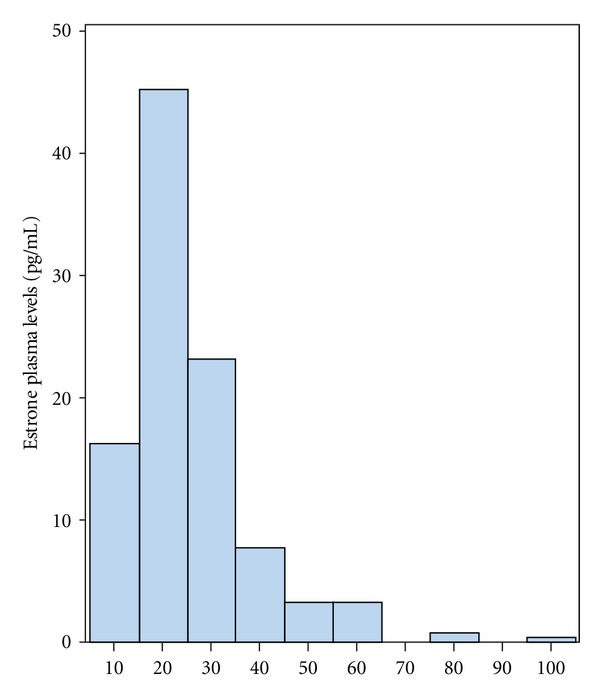
Histogram of estrone plasma levels.

**Figure 2:**
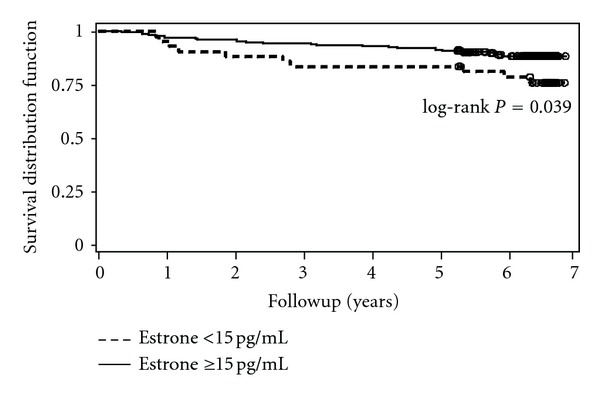
Kaplan-Meier curve for all causes of deaths according to estrone levels (<15 pg/mL versus ≥15 pg/mL).

Baseline clinical and laboratory characteristics of the patients and group comparisons of estrone levels of <15 and ≥15 pg/mL are shown in Table 1. Estrone histogram is shown in Figure 1. Mean estrone levels were, respectively, 10.8 ± 2.1 and 27.6 ± 12.9 pg/mL (*P* < 0.001). Women with a low estrone level were older (72 ± 7.5 versus 69.2 ± 6.7 years; *P* = 0.016), thinner (27.9 ± 4.6 versus 30 ± 5.4 kg/m^2^; *P* = 0.020), and had less hypertension (95% versus 88%; *P* = 0.036), diabetes (37% versus 62%; *P* = 0.003), and lower triglyceride (148.9 ± 63.1 versus 178.5 ± 123.8 mg/dL; *P* = 0.023), glucose (107.5 ± 32.8 versus 137.3 ± 59.5 mg/dL; *P* = 0.002), and C-reactive protein levels (0.45 ± 0.55 versus 0.70 ± 1.31 mg/dL; *P* = 0.045). Women with BMI ≥30 kg/m^2^ were younger (70.5 ± 7.4 versus 68.7 ± 6.3; *P* = 0.046) and had more hypertension (92% versus 99%; *P* = 0.009), less diabetes (52% versus 31%; *P* = 0.001), and lower HDL (54.3 ± 13.2 versus 50.9 ± 11.3 mg/dL; *P* = 0.033) and LDL (117 ± 35.8 versus 107 ± 35.6 mg/dL; *P* = 0.029). Estrone was greater in patients with BMI ≥30 kg/m^2^ (22.4 ± 9.9 versus 28.2 ± 16.4; *P* = 0.001) (Table 2). Death was associated with higher age (69.2 ± 6.9 versus 72.6 ± 7.2 years; *P* = 0.010) and almost significant higher prevalence of hypertension (96% versus 88%; *P* = 0.051) and LDL plasma levels (111.2 ± 37.1 versus 121.3 ± 25.2 mg/dL; *P* = 0.054) (Table 3). There were 32 deaths, 27 (84%) attributed to cardiovascular disease (23 to CAD and 4 to stroke), 3 to pneumonia, and 2 to cancer (1 lung and 1 bone). The Kaplan-Meier survival curve showed a significant trend (*P* = 0.039) of greater all-cause mortality in women with low estrone levels (<15 pg/mL) (Figure 2). Three models of Cox regression analysis were done: model 1 was adjusted for age, body mass index, hypertension, diabetes, dyslipidemia, family history, and estrone; model 2 was adjusted for body mass index, hypertension, diabetes, dyslipidemia, family history, and estrone; model 3 was adjusted for body mass index, diabetes, dyslipidemia, family history, and estrone. In model 1, age (OR = 1.05 [95%CI: 1.00–1.11]; *P* = 0.016) was the only independent predictor of all-cause mortality; in model 2, hypertension (OR = 2.44 [95%CI: 0.83–7.20]; *P* = 0.030); in model 3, estrone (OR = 0.45 [95%CI: 0.21–0.95]; *P* = 0.038) (Table 4).

## 4. Discussion

This study of long-term followup showed that serum estrone level <15 pg/mL was an independent risk factor for increased mortality from all causes in postmenopausal women with CHD or at high risk for CAD. Almost all deaths (84%) were from cardiovascular origin and from myocardial infarction. The current study confirmed the possible beneficial effects of estrone suggested by our previous study of short-term followup in the same population [9]. However, the association of estrone and CAD is unknown. Few studies have analyzed this association, and they have not shown any correlation between estrone plasma levels and CAD [16–18]. In a recent nested case-control study, estrone levels did not correlate with the incidence of CAD [19].

Estrone is the main endogenous estrogen in postmenopausal women. Nevertheless, estrone effects on the vascular system are significantly lower than estradiol mainly present in the premenopausal period [20]. Appropriate physiological levels of estrone in postmenopausal women may therefore bring additional benefits in cardiovascular protection. In postmenopausal women, a reduction occurs in the number and activity of estrogen receptors in tissues, and therefore more potent endogenous or exogenous estrogens are not necessary in this period. Likewise, the doses of exogenous estrogens are not physiological and are generally above the baseline needs. Estrogen therapy significantly increases the serum levels of estrone far above the levels observed in postmenopausal women. Estrone is also the main precursor of the small amount of estradiol still present in postmenopausal women. Estrone can then be the most suitable endogenous estrogen during the postmenopausal period for maintenance of basic physiological needs at this time. The estrone in postmenopausal women is produced mainly in adipose tissue, and obese women have higher serum estrone. However, obesity is associated with metabolic syndrome characterized by alterations in glucose metabolism, lipids, and presence of hypertension [21]. Inflammatory markers, such as CRP and leptin, are also increased in obese individuals. CRP is also increased in postmenopausal women who use hormone replacement therapy. In our study, we observed increased serum levels of CRP in the group of women with estrone ≥15 pg/mL. However, this increase may be due to the higher prevalence of hypertension, diabetes, serum triglycerides, and glucose in this group, suggesting a pattern of metabolic syndrome that is known to increase CRP. Being estrone protective of the cardiovascular system, the increased production of estrone in obese women may be a feedback mechanism for protecting the cardiovascular system in this population. In our study, estrone ≥15 pg/mL may be a factor to explain the lower mortality despite the high risk profile for cardiovascular patients. Higher estrone levels remained an independent variable even after adjustment for body mass index and prevalence of diabetes and dyslipidemia. The action of estrone in the metabolism of glucose and lipid is still controversial [22–25], but direct and indirect beneficial effects similar to those observed for estradiol, but of lesser intensity, may also occur with estrone in these 2 pathways. The prevalence of risk factors for CAD was significantly higher in our population. Diabetes was more prevalent in women with estrone levels ≥15 pg/mL (62% versus 37%, *P* = 0.003), but even with a worse cardiovascular risk profile [26], this was the group that had fewer deaths. In conclusion, serum estrone levels ≥15 pg/mL were associated with lower mortality from all causes in postmenopausal women with CHD or at high risk for CAD. Therefore, we hypothesized that the better prognosis was due to the possible protective effects of increased physiological levels of estrone. Nevertheless, this hypothesis should be validated by a large prospective study.

## Figures and Tables

**Table 1:** Baseline clinical characteristics and comparison of estrone <15 pg/mL and estrone ≥15 pg/mL in 251 patients.

	Baseline *N* = 251	Estrone <15 pg/mL, *N* = 43 (17%)	Estrone ≥15 pg/mL, *N* = 208 (83%)	*P*
Age (years)	69.7 ± 7.0	72 ± 7.5	69.2 ± 6.7	0.016
Body mass index (kg/m^2^)	29.7 ± 5.3	27.9 ± 4.6	30 ± 5.4	0.020
Hypertension	238 (95)	38 (88)	200 (96)	0.036
Diabetes	144 (57)	16 (37)	128 (62)	0.003
Dyslipidemia	233 (93)	40 (93)	193 (93)	0.956
Family history	170 (68)	31 (72)	139 (67)	0.641
Coronary artery disease	189 (75)	35 (81)	154 (74)	0.308
Smoking	25 (10)	4 (10)	21 (10)	0.837
SBP (mm Hg)	141.8 ± 18.4	137.7 ± 21.2	142.6 ± 17.7	0.110
DBP (mm Hg)	83 ± 9.9	80.7 ± 10.8	83.5 ± 9.6	0.092
Triglycerides (mg/dL)	173.4 ± 116.1	148.9 ± 63.1	178.5 ± 123.8	0.023
Total cholesterol (mg/dL)	199.3 ± 41.1	200.7 ± 40.7	199 ± 41.2	0.808
HDL cholesterol (mg/dL)	52.8 ± 12.5	54.8 ± 13.1	52.4 ± 12.3	0.247
LDL cholesterol (mg/dL)	112.7 ± 36	116.1 ± 37.8	112 ± 35.7	0.496
Glucose (mg/dL)	132.2 ± 56.9	107.5 ± 32.8	137.3 ± 59.5	0.002
C reactive protein (mg/dL)	0.65 ± 1.22	0.45 ± 0.55	0.70 ± 1.31	0.045
*Estrone (pg/mL)*	24.9 ± 13.4	10.8 ± 2.1	27.6 ± 12.9	<0.001

SBP: systolic blood pressure; DBP: diastolic blood pressure.

**Table 2:** Baseline clinical characteristics and comparison of body mass index (BMI) <30 kg/m^2^ and ≥30 kg/m^2^ in 246 patients.

	IMC <30 kg/m^2^ (*N* = 141)	IMC ≥30 kg/m^2^ (*N* = 105)	*P*
Age (years)	70.5 ± 7.4	68.7 ± 6.3	0.046
Hypertension	129 (92)	104 (99)	0.009
Diabetes	73 (52)	33 (31)	0.001
Dyslipidemia	131 (93)	97 (92)	0.667
Family history	94 (67)	76 (72)	0.337
Coronary artery disease	36 (26)	24 (23)	0.629
Smoking	14 (10)	11 (10)	0.888
SBP (mm Hg)	139.6 ± 19.8	144.6 ± 15.9	0.026
DBP (mm Hg)	81.8 ± 10.4	84.67 ± 8.9	0.020
Triglycerides (mg/dL)	160.2 ± 81.0	190.9 ± 149.3	0.055
Total cholesterol (mg/dL)	202.9 ± 41.7	194.7 ± 39.9	0.116
HDL cholesterol (mg/dL)	54.3 ± 13.2	50.9 ± 11.3	0.033
LDL cholesterol (mg/dL)	117.0 ± 35.8	107.0 ± 35.6	0.029
Glucose (mg/dL)	128.6 ± 57.2	136.9 ± 56.3	0.249
C reactive protein (mg/dL)	0.25 ± 0.13	0.63 ± 0.57	0.779
*Estrone (pg/mL)*	22.4 ± 9.9	28.2 ± 16.4	0.001

SBP: systolic blood pressure; DBP: diastolic blood pressure.

**Table 3:** Baseline clinical characteristics and comparison of 246 patients by survival status (32 deaths).

	Alive (*N* = 214)	Death (*N* = 32)	*P*
Age (years)	69.2 ± 6.9	72.6 ± 7.2	0.010
Body mass index	29.7 ± 5.2	28.3 ± 5.7	0.157
Hypertension	205 (96)	28 (88)	0.051
Diabetes	93 (43)	13 (41)	0.763
Dyslipidemia	200 (93)	28 (88)	0.227
Coronary artery disease	54 (25)	6 (19)	0.426
Family history	151 (71)	19 (59)	0.202
Smoking	23 (11)	2 (6)	0.432
SBP (mm Hg)	141.4 ± 18.2	143.1 ± 19.7	0.612
DBP (mm Hg)	82.9 ± 9.8	82.8 ± 10.2	0.938
Triglycerides (mg/dL)	172.2 ± 119	170.6 ± 101	0.908
Total cholesterol (mg/dL)	198.4 ± 41.9	204 ± 33.9	0.468
HDL cholesterol (mg/dL)	53.3 ± 12.8	50.6 ± 10.7	0.251
LDL cholesterol (mg/dL)	111.2 ± 37.1	121.3 ± 25.2	0.054
Glucose (mg/dL)	132.6 ± 56.4	131.3 ± 64.5	0.904
C reactive protein (mg/dL)	0.62 ± 1.04	0.85 ± 2.12	0.548
*Estrone (pg/mL)*	25.1 ± 13.6	21.6 ± 11.5	0.166

SBP: systolic blood pressure; DBP: diastolic blood pressure.

**Table 4:** Cox regression multivariate analysis for different models of variable adjustments.

Model	Variable	Hazard ratio	95% Confidence interval	*P*
1	Age	1.05	1.00–1.11	0.016
2	Hypertension	2.44	0.83–7.20	0.030
3	Estrone	0.45	0.21–0.95	0.038

Model 1: adjusted for age, body mass index, hypertension, diabetes, dyslipidemia, family history, and estrone; 2: adjusted for body mass index, hypertension, diabetes, dyslipidemia, family history, and estrone; 3: adjusted for body mass index, diabetes, dyslipidemia, family history, and estrone.
